# Editorial: Highlights in psychopathology: Mental health among young adults

**DOI:** 10.3389/fpsyg.2023.1163957

**Published:** 2023-03-22

**Authors:** Paolo Meneguzzo, Alessandra Maria Passarotti, Sibel Demir Kaçan

**Affiliations:** ^1^Department of Neuroscience, University of Padova, Padua, Italy; ^2^Padova Neuroscience Center, University of Padova, Padua, Italy; ^3^Department of Psychology, University of Illinois, Chicago, IL, United States; ^4^Education Faculty Science Education Department, Ondokuz Mayıs University Sciences, Samsun, Türkiye

**Keywords:** psychopathology, mental health, adolescent and youth, young adults, wellbeing, psychology, education, psychiatry

A large portion of all behavioral and emotional disorders have an onset in early adolescence and young adulthood, increasing the risk of impaired functioning and mental health in adulthood by two to three times (Pine et al., 1998; Kessler et al., 2007). Even when symptoms are below a clinical threshold, mood and anxiety disorders are associated with a large disease burden in youth, with many negative physical, social, behavioral, and health outcomes (Kessler et al., 2012; Shah et al., 2020). Psychopathological trajectories in youth have been connected to different features that might be related to neurobiological vulnerabilities (Beauchaine, 2015; Langenecker et al., 2018), an adverse environment (Hong et al., 2022), as well as their interactions (Teicher and Samson, 2013; Meneguzzo et al., 2022). Recently, the global burden effect of the COVID-19 Pandemic has corroborated how youths are vulnerable to environmental changes and challenges, external adversity, and abrupt changes in life trajectories (Carroll et al., 2021). Researchers have also shown that the relationship between these factors is quite complex. In the past 3 years the Pandemic has had profound effects on youth mental health, with reports of an increase in mood disorder rates, and requests for help with several psychiatric conditions: depression, anxiety, eating disorders and maladaptive behaviors (Konstantopoulou and Raikou, 2020; Liu et al., 2020; Monteleone et al., 2021). Moreover, suicidal ideation and suicidal behaviors in youths are a significant health problem world-wide (Lyu et al., 2021), that has only increased with the Pandemic (Manchia et al., 2022). For all these reasons, the literature needs to focus on this critical and transitional phase from childhood to adulthood and examine both environmental and biological vulnerabilities due to the possible presence of new stressors—such as the recent Pandemic (Hankin, 2012; Langenecker et al., 2014; Kujawa and Burkhouse, 2017).

The four papers in this Research Topic cover a few important psychological constructs in young adults, touching on psycho-social functioning related to social media, insomnia and studying behaviors, suicidal ideation and COVID-related challenges. We highlight below the key points for each article.

The article by He et al., titled “*Common predictive factors of social media addiction and eating disorder symptoms in female college students: State anxiety and the mediating role of cognitive flexibility/sustained attention*” evaluated relations between social media addiction and eating psychopathology in a sample of 216 Chinese female undergraduate students. Their findings showed a significant correlation between social media addiction and eating disorder symptoms. Moreover, the results showed the mediating roles of cognitive flexibility and sustained attention in state anxiety, social media addiction, and eating disorders. Importantly, these results might reflect the mediating role of specific cognitive processes such as cognitive flexibility and sustained attention in mitigating anxiety and reducingeating psychopathology and social media addiction.

The article by Alshammari et al. titled “*Examining bedtime procrastination, study engagement, and studyholism in undergraduate students, and their association with insomnia*” evaluated a sample of 495 university students from several regions in Saudi Arabia, to examine relationss between insomnia, study engagement, studyholism (i.e., compulsive overstudying), and bedtime procrastination. One of the main points raised by the authors was the need to increase awareness of insomnia problems in youths, and its relationships with the psychopathological aspects of procrastination in studying. Moreover, gender analysis showed significant gender differences, with female participants reporting higher studyholism and bedtime procrastination than male participants. Higher levels of study holism were found in older students compared to younger ones. Finally, the results indicated that insomnia could be positively predicted by levels of studyholism and bedtime procrastination. These findings highlight a need for fostering insomnia awareness programs and educational workshops to teach college students healthier sleep habits, especially in females and students more vulnerable to stress.

The article by Xin et al., “*Relationships between negative life events and suicidal ideation among youth in China: The direct and moderating effects of offline and online social support from gender perspective*” collected questionnaire-based data on negative life events, social support, and suicidal ideation from 2,018 young adults. Participants were high school and university students from Northwestern China. The results showed the direct and moderating effects of offline and online support on suicidal ideation among youths. They also suggested that offline social support had a significant direct effect on suicidal ideation across genders, with differences between male and female participants. Specifically, in young males, offline social support moderated the relationship between negative life events and suicidal ideation. In contrast, the moderating effects in female youth were effective for all negative events. Moreover, online social support had a significant direct effect only on female suicidal ideation. At the same time, it moderated the relationships between negative life events and suicidal ideation for all participants. In sum, these interesting findings stress the need for more research exploring suicide across various cultures and societies, and for gender-informed suicide prevention and intervention.

Finally, the article by Xu et al., **“***College students' creativity during the COVID-19 pandemic: The mediating effect of post-traumatic growth and the moderating role of psychological resilience***”** focused on the effects of the COVID-19 pandemic on a sample of 475 university Chinese students. Their findings provided evidence that intrusive rumination affects creativity, both directly and indirectly, through post-traumatic growth. Psychological resilience played a moderating role between intrusive rumination and creativity. There was also a stronger correlation between intrusive rumination and post-traumatic growth when levels of psychological resilience were higher. These results were discussed in terms of the roles they might play in the worldwide public health emergency of the COVID-19 pandemic. Finally, these results might inform future efforts to improve adolescents' resilience to external stressors.

In conclusion, the current Research Topic generated an exciting and relevant collection of studies focused on evaluating various areas of current vulnerability in youth. They explored social support, studyholism, and social media addiction. More research should include a specific focus on adolescence because internal and external challenges affect it significantly. More tailored and specific interventions to reduce the effects of adverse events and improve psychological resilience in adolescence and young adulthood are fundamental for the wellbeing of future generations.

## Author contributions

PM wrote and prepared the first draft of the manuscript. AP and SD revised and supervised the first draft. All authors have approved the submitted version.
